# Serum amyloid A in HFpEF and cardiometabolic diseases

**DOI:** 10.1007/s00395-025-01150-9

**Published:** 2025-11-28

**Authors:** Luo Liu, Rongling Wang, Stefano Strocchi, Tolga Eroglu, Natasha Nambiar, Sarah V. Liévano Contreras, Saskia A. Diezel, Gabriele G. Schiattarella

**Affiliations:** 1 Department of Cardiology, Angiology and Intensive Care Medicine, Deutsches Herzzentrum der Charité (DHZC), Max Rubner Center for Cardiovascular Metabolic Renal Research (MRC), Charité-Universitätsmedizin Berlin, Berlin, Germany; 2 DZHK (German Centre for Cardiovascular Research), Partner Site Berlin, Berlin, Germany; 3 Translational Approaches in Heart Failure and Cardiometabolic Disease, Max Delbrück Center for Molecular Medicine in the Helmholtz Association (MDC), Berlin, Germany; 4 Friede Springer Cardiovascular Prevention Center at Charité – Universitätsmedizin Berlin, Berlin, Germany; 5 Experimental and Clinical Research Center (ECRC), a Cooperation of Charité Universitätsmedizin Berlin and Max Delbruck Center for Molecular Medicine (MDC), Berlin, Germany; 6 Division of Cardiology, Department of Advanced Biomedical Sciences, Federico II University, Naples, Italy

**Keywords:** HFpEF, MASLD, Metabolic dysfunction, Inflammation, Serum amyloid A

## Abstract

Heart failure with preserved ejection fraction (HFpEF) accounts for more than half of all heart failure cases, and its prevalence is projected to rise further. Among its heterogeneous subtypes, cardiometabolic HFpEF, which is driven by metabolic dysfunction, represents a globally predominant form. Recent advances in preclinical models have highlighted metabolic disturbances and systemic inflammation as key contributors to HFpEF pathogenesis. While much attention has focused on classical inflammatory mediators such as interleukin-6 (IL-6) and tumor necrosis factor-α (TNF-α), the full spectrum of upstream inflammatory drivers and the therapeutic strategies targeting inflammation in cardiometabolic HFpEF remain incompletely defined. Among emerging candidates, serum amyloid A (SAA) family proteins, highly inducible acute-phase proteins, have attracted growing attention due to their elevated levels in chronic metabolic diseases. Here, we summarize clinical associations between elevated SAA levels and major cardiometabolic conditions—including obesity, diabetes, metabolic dysfunction-associated steatotic liver disease (MASLD, formerly NAFLD), and hypertension—and discuss potential mechanisms based on preclinical studies. We place particular emphasis on the known and potential pathogenetic role of SAA in cardiometabolic HFpEF, where it may contribute to systemic inflammation, endothelial dysfunction, and myocardial fibrosis. Overall, this review aims to advance understanding of SAA in HFpEF and cardiometabolic disease, and to support translational efforts toward improved diagnosis and treatment.

## Introduction

Heart failure with preserved ejection fraction (HFpEF) is a complex clinical syndrome that has emerged as a major global health concern, characterized by high morbidity and mortality. It affects approximately 3 million people in the United States and up to 32 million worldwide, with its prevalence expected to rise as the population continues to age [126]. In contrast to heart failure with reduced ejection fraction (HFrEF), HFpEF remains largely devoid of effective evidence-based therapies, and its rising prevalence underscores a critical unmet clinical need. Over the past decade, low-grade chronic inflammation driven by metabolic comorbidities—commonly referred to as meta-inflammation—has been increasingly recognized as a central contributor to HFpEF pathophysiology, highlighting inflammation as a promising therapeutic target [137]. A wide range of inflammatory mediators—such as interleukin-6 (IL-6), tumor necrosis factor-α (TNF-α), and myeloperoxidase (MPO)—have been implicated in the pathogenesis of HFpEF. These molecules not only serve as biomarkers of systemic inflammation but also actively contribute to endothelial dysfunction, myocardial remodelling, and impaired exercise tolerance. While most studies have focused on these cytokines, the full spectrum of inflammatory drivers and their translational relevance in HFpEF has yet to be fully delineated. As interest shifts toward less-characterized mediators, acute-phase proteins such as serum amyloid A (SAA) have garnered renewed attention.

SAA proteins are highly conserved acute-phase reactants that respond rapidly to systemic stressors such as infection, trauma, or malignancy [79]. While levels are typically < 3 mg/L in healthy individuals, they can rise up to 1000-fold in acute inflammation and approximately fivefold in chronic low-grade inflammation conditions [107]. Emerging evidence has implicated chronically elevated SAA in the pathogenesis of several cardiometabolic diseases, including obesity, type 2 diabetes mellitus (T2DM), MASLD and hypertension [18, 69, 105, 125, 168]. These metabolic disorders commonly coexist in individuals with the cardiometabolic phenotype of HFpEF [62, 137, 159], raising the possibility that SAA contributes to HFpEF pathogenesis. Indeed, recent clinical studies have reported increased circulating SAA1 levels in patients with HFpEF [35, 132]. It is worth noting that HFpEF is still predominant in elderly, and aging itself exacerbates the state of chronic low-grade inflammation through a mechanism known as inflammaging [96]. SAA levels have been shown to increase significantly with age [84]. This aging-related inflammatory burden may act synergistically with metabolic inflammation to promote the development of HFpEF, in which SAA could play an important role.

However, direct evidence supporting a causal or mechanistic role for SAA in human HFpEF remains limited. In this review, we examine current knowledge regarding the role of SAA in cardiometabolic diseases and explore its potential contribution to HFpEF pathophysiology. We also discuss the diagnostic and therapeutic implications of targeting SAA in this increasingly prevalent condition.

## SAA subtypes, receptors and biological function

The biological function of SAA is mediated by its molecular structure, isoform diversity, and receptor interactions. SAA proteins constitute a family of small, highly conserved acute-phase proteins comprising 103–104 amino acids with strong sequence homology across vertebrate species [155]. In humans and mice, the SAA gene cluster is located on chromosome 11p and chromosome 7, respectively [140]. The family includes four genes (SAA1–SAA4), among which SAA1 and SAA2 encode the inducible acute-phase isoforms that are markedly upregulated in response to inflammatory stimuli [55]. Additionally, SAA1 directly binds to cholesterol and saturated fatty acids, implicating a regulatory role in lipid trafficking and lipotoxicity. Although SAA1 and SAA2 share over 93% sequence homology [19], subtle amino acid differences may underlie functional divergence. Both are strongly induced by cytokines (IL-6, IL-1β, TNF-α), but SAA1 shows stronger upregulation in monocytes and macrophages following LPS or steroid stimulation [72]. Both isoforms engage common receptors including FPR2, TLR2/4, and SR-B1, but direct comparisons of their binding affinities are lacking. Evidence more consistently links SAA1 to activation of pro-inflammatory signaling and to chemotactic or lipid metabolic effects [6, 46, 58, 69], whereas the precise roles of SAA2 remain less well defined. Collectively, most experimental studies have focused on SAA1 or mixed SAA1/2 preparations, with relatively few dissecting SAA2-specific functions. Unlike SAA1 and SAA2, the SAA3 gene has become a non-functional pseudogene in humans and does not produce a biologically active protein [155]. In mice, Saa3 is more divergent than Saa1 and Saa2 and is primarily expressed extrahepatically, being strongly inducible in adipocytes and macrophages under inflammatory conditions [108, 134]. Its expression has also been reported in lungs, intestines, and kidneys [110, 127]. SAA4 is a constitutively expressed isoform of the SAA family, mainly synthesized in the liver and largely unresponsive to inflammation [27, 155]. It has been implicated in High-density lipoproteins (HDL) remodeling, lipid metabolism [28], and possibly thrombotic risk [42], although its precise biological functions remain poorly defined. During acute inflammation, SAA is primarily synthesized by hepatocytes. In contrast, under chronic inflammatory conditions, extrahepatic production occurs in adipose tissue, intestinal epithelial cells, and macrophages within inflamed tissues [32]. In accordance with scientific nomenclature standards, “SAA” refers to the human proteins in this review, while “Saa” denotes their murine counterparts.

**Fig. 1:**
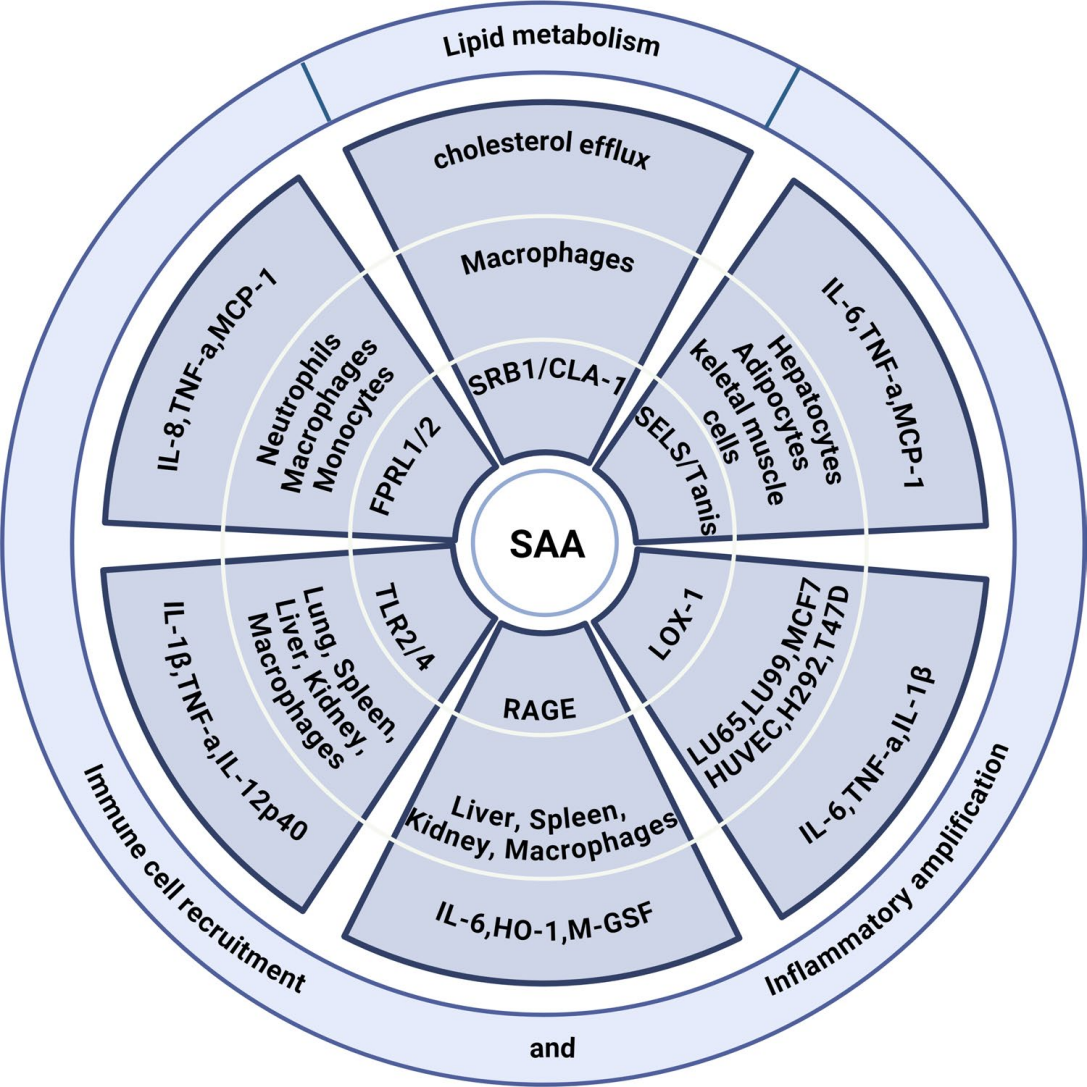
SAA-driven signaling and biological functions. This figure summarizes the receptor-mediated signaling landscape and functional outputs of serum amyloid A (SAA). The central layer depicts SAA as a pleiotropic inflammatory mediator that interacts with multiple cell-surface receptors, including SRB1/CLA-1, FPRL1/2, SELS (Tanis), RAGE, TLR2/4, and LOX1. These interactions activate distinct intracellular pathways, resulting in the production of proinflammatory cytokines (e.g., IL-1β, IL-6, TNF-α, MCP-1), stress-related mediators (e.g., HO-1, M-CSF), and alterations in lipid handling (e.g., impaired cholesterol efflux). The outer ring categorizes these downstream effects into three major biological domains: lipid metabolism, immune cell recruitment and inflammatory amplification. Together, this schematic highlights the diverse and multifaceted roles of SAA in shaping inflammatory and metabolic responses

SAA exerts its biological effects through interaction with multiple cell surface receptors, including formyl peptide receptor-like 1 and 2 (FPRL1/2), toll-like receptor 2 and 4 (TLR2, TLR4), receptor for advanced glycation end products (RAGE), lectin-like oxidized low-density lipoprotein receptor-1 (LOX-1), scavenger receptor class B type I (SR-BI; CLA-1 in mice), and selenoprotein S (SELS, Tanis in animal models) [32]. These receptors mediate diverse downstream signaling cascades (Fig. 1). In humans, CLA-1 and its murine ortholog SR-BI mediate SAA-induced cholesterol efflux and activate extracellular signal-regulated kinase (ERK)and p38 mitogen-activated protein kinase (p38/MAPK) despite their short intracellular domains [7, 8, 14]. In macrophages, SR-BI also mediates the uptake of SAA, leading to intracellular processing and formation of extracellular amyloid A fibrils [76]. FPRL1 and FPRL2, as G protein-coupled chemotactic receptors, activate MAPK and nuclear factor kappa B (NF-κB) pathways upon SAA binding, resulting in the release of proinflammatory mediators such as TNF-α, interleukin-8 (IL-8), and monocyte chemotactic protein-1 (MCP-1) [1, 24, 47, 57, 87, 89, 95, 147]. SAA also activates TLR2 and TLR4, which induce phosphorylation of ERK and p38/MAPK and upregulate cytokines, including IL-1β, TNF-α, interleukin-12 subunit p40(IL-12p40), interleukin-1 receptor antagonist (IL-1ra), and IL-10 [22], as well as enhanced nitric oxide (NO) production [135]. TLR2 activation additionally promotes expression of Interleukin-23 subunit p19(IL-23p19) [58] and Granulocyte colony-stimulating factor (G-CSF) [59]. Through engagement of the AGE–RAGE axis, SAA enhances the expression of IL-6, heme oxygenase-1 (HO-1), and macrophage colony-stimulating factor (M-CSF), while binding to LOX-1 further amplifies NF-κB-mediated IL-6 and TNF-α production [75, 94, 130, 166].

Given its ability to engage multiple receptors, SAA can simultaneously activate parallel inflammatory and metabolic signaling networks. Receptor-mediated processes—such as chemotaxis and activation of ERK, p38 MAPK, and NF-κB—are further modulated by the surrounding tissue-specific cytokine environment. Collectively, SAA plays multifaceted roles in host defense, immune cell recruitment, lipid metabolism, and inflammatory amplification.

## SAA in cardiometabolic diseases

### Obesity

SAA expression is closely linked to obesity, one of the key cardiometabolic conditions in which its pathophysiological roles have been most extensively studied. The major clinical associations and mechanistic roles of SAA in key cardiometabolic diseases are summarized in Table 1. Several studies have demonstrated that pro-inflammatory cytokines such as IL-1β, IL-6, and TNF-α can significantly upregulate SAA1 and SAA2 expression in adipose tissue [122], and both SAA1 and SAA2 are markedly elevated in subcutaneous white adipose tissue (WAT) of overweight and obese individuals [67, 143]. Quantitatively, obese individuals exhibit approximately a six-fold increase in SAA expression within subcutaneous WAT compared to lean subjects [124]. Circulating SAA levels in obese populations strongly correlate with body mass index (BMI), total fat mass, and the transcript levels of SAA1 and SAA2 in WAT [86, 124, 177]. Notably, weight loss—achieved through dietary or surgical interventions—consistently reduces both circulating and adipose tissue–derived SAA levels [15, 116, 177]. These reductions are frequently accompanied by declines in other inflammatory markers, such as monocyte MCP-1 and C-reactive protein (CRP) [66]. Despite these associations, the relative adipose contributions to circulating SAA levels in obesity remain unclear, as does SAA’s exact role in chronic adipose inflammation. Nonetheless, current evidence supports its function as a proinflammatory adipokine.

**Table 1:** Representative preclinical and clinical evidence linking SAA to cardiometabolic disease

Disease/type of evidence	Model/population	Condition/intervention	SAA findings	Mechanisms/outcomes	References
Obese (preclinical)	Saa3 knockout C57BL/6N mice	HFHSC diet	Both male and female Saa3−/− mice had reduced liver Saa1 and Saa2 expression in association with reduced plasma SAA	Saa3 deficiency attenuates weight gain (both sexes); in females, additionally reduces adipose inflammation and improves lipid profile	[32]
	Male Swiss Webster mice	HFD + SAA-targeted ASO	SAA-targeted ASO abolished HFD-induced elevation of SAA levels	SAA depletion prevented HFD-induced weight gain, adipose tissue expansion, and macrophage infiltration	[28]
Obese (clinical)	Adipose tissue (n = 12, BMI 23.0–28.7 kg/m^2^)	Separating adipocytes by size (small vs. large)	SAA expression was approximately 19-fold higher in large adipocytes compared with small adipocytes, at both mRNA and protein levels	SAA1 and SAA2 expression were higher in subcutaneous white adipose tissue in people with overweight	[66]
	5 lean, 12 obese normoglycemic, 14 obese (RYGB cohort)	Roux-en-Y gastric bypass (before vs. after)	Circulating SAA levels were higher in obese than in lean subjects; SAA were negatively associated with HDL-cholesterol concentrations	Weight loss after RYGB induced a statistically significant reduction in circulating levels of SAA	[14]
Diabetes (preclinical)	C57Bl/6 mice; 3T3-L1 adipocytes	HFD diet feeding; recombinant A-SAA treatment in vitro	HFD feeding leads to early upregulation of Saa3 in adipose tissue, followed by increased hepatic expression of Saa1 and Saa2; recombinant A-SAA induced pro-inflammatory genes and suppressed insulin-sensitivity genes	Acute-phase Saa as a marker of insulin resistance in mice	[134]
	C57Bl/6 mice; Huh7 hepatoma cells	HFD feeding in mice; PA treatment in vitro; SAA1 silencing (shRNA, ad-shSaa1); NF-κB inhibition (BAY 11–7082)	SAA1/Saa1 expression upregulated in HFD mice and PA-treated cells; silencing SAA1 reduced SOCS3, increased IRS1 and IRS1 phosphorylation, improved 2DG uptake	Silencing SAA1 inhibits palmitate- or high-fat diet induced insulin resistance through suppression of the NF-κB pathway	[159]
Diabetes (clinical)	6 with T2DM, 10 controls	Comparison of SAT vs. VAT adipokine expression with metabolic phenotyping and insulin clamp	VAT from T2DM subjects showed significantly higher SAA mRNA expression compared to normal glucose tolerance	SAA expression closely correlated with fasting glucose	[131]
	27 insulin-treated T2DM patients	Troglitazone 400 mg/day vs placebo, 16 weeks	Baseline SAA levels were elevated in T2DM patients (~ 6.2 mg/L) compared with healthy controls (~ 2.1 mg/L)	Troglitazone treatment decreased circulating SAA concentrations by approximately 35%, with levels reverting to baseline following drug withdrawal	[37]
NAFLD (preclinical)	SAA1/2 knockout C57BL/6J mice	HFD (16 weeks) or WD (8 weeks)	SAA1 expression was significantly upregulated in the liver, paralleled by increased plasma SAA1 concentrations	SAA1 exacerbates hepatic steatosis via TLR4-mediated NF-κB signaling pathway	[68]
	Female BALB/C mice	HFD-fed mice injected with Lv-SAA1-shRNA-EGFP	SAA1 was highly expressed in fatty liver tissue and steatotic hepatocytes	Hepatocytes derived increased SAA1 promotes intrahepatic platelet aggregation and aggravates liver inflammation in NAFLD	[91]
NAFLD (clinical)	30 liver tissues from patients with NAFLD	IHC staining	Relative to controls, both mild and severe NAFLD exhibited higher hepatic SAA1 expression	hepatic SAA1 level was positively correlated with NAFLD activity score and ALT	[68]
Hypertension (preclinical)	Human aortic endothelial cells; rat aortic rings	SAA (0.25–25 μg/ml) ± HDL pretreatment	SAA decreased NO bioavailability and cGMP; induced Ca^2^⁺ influx and superoxide radical anion generation; upregulated TF, NF-κB, IL-8, Arg-1	SAA may promote endothelial dysfunction by modulating NO and l-Arg bioavailability	[161]
Hypertension (clinical)	165 newly diagnosed, untreated stage I–II essential hypertension patients	Cardiac ultrasonography and laboratory measurements	Systolic blood pressure was independently associated with circulating levels of SAA	Altered LV geometry is associated with elevated serum SAA in newly essential diagnosed hypertension	[151]
	323 patients with essential hypertension and without diabetes	6 months treatment with ARBs or ARB/diuretic combination	6 months of ARB or ARB/diuretic therapy significantly reduced serum SAA levels, from 5.09 to 4.09 mg/dL	A smaller decrease in both SAA and hsCRP levels in smokers compared with nonsmokers after therapy	[79]

*HFHSC* high-fat, high-sucrose, cholesterol-rich, *HFD* high-fat diet, *ASO* antisense oligonucleotide, *RYGB* Roux-en-Y gastric bypass, *shRNA* short hairpin RNA, *ad-shSaa1* adenovirus-delivered shRNA targeting SAA1, *PA* palmitate, *SOCS3* suppressor of cytokine signaling 3, *IRS1* insulin receptor substrate 1, *2DG* 2-deoxyglucose, *SAT* subcutaneous adipose tissue, *VAT* visceral adipose tissue, *T2DM* type 2 diabetes mellitus, *NAFLD* non-alcoholic fatty liver disease, *Lv-SAA1-shRNA-EGFP* Lentivirus carrying SAA1-targeting shRNA and EGFP, *IHC* immunohistochemistry, *ALT* alanine aminotransferase, *NO* nitric oxide, *TF* tissue factor, *Arg-1* Arginase-1, *cGMP* cyclic guanosine monophosphate, *NF-κB* nuclear factor kappa-light-chain-enhancer of activated B cells, *ARBs* angiotensin II receptor blockers, *hsCRP* high-sensitivity C-reactive protein

In murine models, Saa3 is selectively expressed in adipocytes and macrophages—two key cell types involved in obesity-associated inflammation [10, 108, 131]—and its expression is consistently elevated in the adipose tissue of obese mice [98, 145]. Supporting these findings, in vitro studies have demonstrated that mRNA and protein levels in adipocytes are upregulated by various metabolic and inflammatory factors, including elevated glucose, fatty acids, and cytokines such as TNF-α, IL-1β, and LPS [31, 98, 144, 170]. Nevertheless, functional data from Saa gene knockout models have shown variable outcomes. One study reported that selective deletion of extrahepatic Saa3 attenuated adipose tissue inflammation and conferred resistance to high-fat diet-induced obesity [33], whereas another study found that Saa3-deficient mice exhibited greater weight gain under similar conditions [5]. Furthermore, triple-knockout mice lacking Saa1, Saa2, and Saa3 showed no significant differences in obesity development or adipose inflammation compared to wild-type controls [68]. These divergent findings may reflect differences in genetic background, gut microbiota composition, dietary regimen, or the specific genes targeted for deletion.

Additional studies have shown that SAA expression positively correlates with adipocyte size in obese individuals [123]. In vitro silencing of Saa3 in preadipocytes impairs adipogenesis and leads to smaller fat depots when implanted into nude mice [156]. Antisense oligonucleotide (ASO)–mediated knockdown of Saa similarly reduces adipose tissue expansion and inflammation in murine models [29]. Elevated Saa3 expression in visceral adipose tissue has also been associated with increased macrophage infiltration and a shift toward a proinflammatory cytokine milieu [134]. In this inflammatory context, macrophage-derived cytokines enhance SAA expression in adipocytes [123], while SAA, in turn, stimulates macrophages to produce IL-6, IL-8, TNF-α, and MCP-1 [167]. Given the central role of macrophages in coordinating adipose inflammation, SAA likely serves as a key modulator of macrophage–adipocyte crosstalk, contributing to the persistence of low-grade inflammation in obesity. It is important to note that much of the mechanistic insight derives from murine models investigating Saa3, which is a pseudogene in humans. While the phenotypes and signaling pathways identified in mouse Saa3 models may inform hypotheses regarding SAA1/2 function in human adipose tissue, these findings require validation in human-relevant systems.

### Diabetes

Beyond its link to obesity, SAA has been implicated in the pathogenesis of T2DM [37, 51, 154]. In one study, omental adipose tissue from patients with T2DM exhibited a threefold increase in SAA mRNA expression compared to non-diabetic controls, and expression levels correlated closely with fasting glucose concentrations [133]. A large population-based study of 756 men aged over 70 further demonstrated a significant association between serum SAA levels and diabetic status [60]. In support of this association, several antidiabetic agents—including metformin, glipizide, rosiglitazone, insulin, and acarbose—have been shown to reduce circulating SAA levels in individuals with T2DM [37, 38, 167]. However, given that most individuals with T2DM are also obese, disentangling the contributions of SAA from adiposity remains challenging. Notably, some studies support this possibility: in one cohort of individuals with T2DM and BMI-matched healthy controls, SAA levels remained significantly elevated in the diabetic group despite similar BMI (~ 24) [99]. Another study of 134 patients with T2DM reported persistent associations between SAA levels and HbA1c and HOMA-IR after adjusting for age, sex, and BMI [91]. Moreover, treatment of overweight or obese individuals with rosiglitazone for 12 weeks led to a 37% reduction in circulating SAA levels without changes in body weight, accompanied by decreased SAA secretion from subcutaneous adipose tissue explants [167]. Nevertheless, not all studies have reported comparable findings; for instance, one investigation found no difference in SAA levels between insulin-sensitive individuals and patients with T2DM [121]. Thus, while SAA closely associates with T2DM, its potential role as an independent contributor to disease pathophysiology remains uncertain.

Mechanistic insights from experimental models support this clinical link. In murine models, high-fat diet (HFD) feeding leads to early upregulation of Saa3 in adipose tissue, followed by increased hepatic expression of Saa1 and Saa2, in parallel with the development of insulin resistance and rising serum Saa levels [136]. Early studies demonstrated that recombinant SAA (rSAA) significantly reduced GLUT4 mRNA expression during preadipocyte differentiation leading to impaired glucose transport and attenuating insulin responsiveness [43]. Subsequent work showed that in palmitate- or HFD-induced models, Saa1 upregulation inhibited IRS-1 signaling via NF-κB activation, contributing to insulin resistance [161]. Receptor-level evidence further supports these effects: overexpression of the SAA receptor Tanis in hepatocytes impairs insulin-stimulated glucose uptake and glycogen synthesis [73], while SELS expression in adipose tissue correlates positively with glycemic control in humans with T2DM [169]. Together, these findings indicate that SAA promotes insulin resistance through receptor-mediated mechanisms, most likely involving NF-κB-driven disruption of the IRS-1/PI3K/Akt signaling cascade, a hypothesis that requires validation in clinical settings to establish its translational relevance.

Beyond diet-induced obesity models, evidence from genetic and chemically induced diabetes models also implicates SAA in diabetic pathophysiology. Leptin-deficient ob/ob mice displayed elevated Saa3 and Saa4 mRNA in adipose tissue, while Saa3 upregulation in adipose tissue was also observed in streptozotocin (STZ)-induced diabetes [98, 145]. In db/db mice, serum Saa concentrations were markedly increased, paralleling lipid accumulation across multiple organs [101]. Collectively, these findings suggest that SAA elevation is not restricted to obesity-driven forms but may act as a more general inflammatory mediator in diabetes.

### NAFLD/MASLD

Nonalcoholic fatty liver disease (NAFLD), recently redefined as metabolic dysfunction–associated steatotic liver disease (MASLD), characterized by triglyceride accumulation in hepatocytes, can progress to steatohepatitis (NASH/MASH) and hepatic fibrosis. Clinical studies have shown that serum SAA levels are elevated two to threefold in patients with NASH compared to age-matched healthy controls [173], a finding corroborated by similar increases in Saa expression in murine models of NAFLD [69, 92]. Despite these associations, the clinical utility of SAA as a biomarker for NAFLD remains limited due to insufficient specificity. Nonetheless, recent findings have begun to elucidate its potential mechanistic role. Preclinical data suggest that genetic deletion or hepatic knockdown of Saa1/2 enhances energy expenditure and attenuates high-fat diet (HFD)-induced steatosis, metabolic dysfunction, and hepatic inflammation. These protective effects have been partially attributed to suppression of TLR4-NF-κB signalling, a pathway implicated in hepatic lipid accumulation [69]. Beyond lipid metabolism, SAA1 has also been implicated in hepatocyte–platelet interactions. In one murine study, increased hepatocyte Saa1 expression promoted intrahepatic platelet adhesion and activation, thereby exacerbating liver inflammation in NAFLD [92]. In parallel, hepatic secretion of Saa1 mediated by SURF4 was shown to activate hepatic stellate cells (HSCs), contributing to fibrogenesis in a mouse model of liver fibrosis [158]. Additionally, SAA has been proposed as a biomarker of early-stage fibrosis in certain liver diseases [39]. Further evidence of Saa’s involvement in NAFLD progression comes from studies in hypercholesterolemic mice lacking IL-1α or IL-1β, which are key cytokines required for the transition from steatosis to NASH and fibrosis [148]. Given that IL-1β is a potent inducer of hepatic SAA expression, these findings suggest that SAA may act as a downstream effector of IL-1β-mediated hepatic injury. Although a variety of drugs and therapeutic approaches targeting FXR agonists, GLP-1 receptor agonists, or PPAR agonists are implicated in treatment of NASH, whether and how these treatments affect SAA levels remains to be further explored.

### Hypertension

Beyond its role in metabolic disorders, SAA has been increasingly implicated in chronic inflammation. Multiple cross-sectional and longitudinal studies support an association between systemic inflammation and elevated blood pressure, potentially mediated by imbalances between vasoconstrictors and vasodilators, enhanced thrombogenicity, and direct vascular effects of inflammatory mediators [48]. As a result, low-grade inflammation has emerged as a contributor to the pathogenesis of hypertension, and targeting inflammatory pathways may hold therapeutic potential [176]. In both a multicenter cohort of approximately 1000 individuals with hyperlipidemia and a separate large cohort of newly diagnosed hypertensive patients without diabetes, systolic blood pressure (SBP) was independently associated with circulating levels of SAA [153, 157]. Notably, 6 months of angiotensin receptor blocker (ARB) therapy significantly reduced circulating SAA concentrations [80]. In another study of hypertensive patients with comorbid diabetes, SAA levels were inversely correlated with the small artery media-to-lumen ratio, suggesting a link between elevated SAA and adverse microvascular remodeling [146].

Mechanistic insights from in vitro studies further explore the potential mechanism of SAA in hypertension. SAA may impair vascular homeostasis by simultaneously suppressing vasodilatory signaling and augmenting pro-constrictive pathways through its inflammatory actions. In human aortic endothelial cells (HAECs), SAA reduces NO bioavailability, likely via enhanced superoxide generation [163]. Consistent findings have been reported in porcine endothelial cells, where SAA downregulated eNOS expression and impaired NO production through activation of c-Jun N-terminal kinase (JNK), ERK1/2, and NF-κB signaling pathways [160]. Although direct evidence linking SAA to vasoconstrictors such as endothelin-1 (ET-1) is limited, it is well established that inflammatory cytokines—including IL-1β [171], TNF-α [64], and IL-6 [165]—stimulate ET-1 expression. Given that SAA potently induces these cytokines, it is plausible that SAA indirectly promotes vasoconstrictor upregulation via inflammation-driven ET-1 production. Together, these findings suggest that SAA may disrupt vascular homeostasis by shifting the balance from vasodilation toward vasoconstriction through both direct suppression of NO signaling and indirect upregulation of ET-1 via inflammatory mediators.

In addition to its functional effects on vascular tone, SAA may also promote vascular stiffness and structural remodeling. In an in vitro study using rat aortic smooth muscle cells (RASMCs), recombinant SAA induced a phenotypic switch from a contractile to a synthetic state, characterized by reduced expression of α-SMA and SM22α, and a dose-dependent increase in the mRNA expression of extracellular matrix (ECM) synthesis-related markers, including elastin, collagen I, collagen III, matrix metalloproteinase (MMP2), and MMP9 [175]. These phenotypic changes were accompanied by increased cell proliferation and migration, mediated through p38 MAPK signaling. In line with this, another study demonstrated that Saa1 promotes MMP expression both in vascular smooth muscle cells and in macrophages [162]. Furthermore, SAA was shown to activate TLR2 signaling, upregulate MMP9, and downregulate tropoelastin expression in RASMCs, thereby impairing elastin fiber formation and contributing to extracellular matrix remodeling [139]. Primary hypertension is also closely linked to atherosclerosis (AS), and these conditions may act synergistically to perpetuate vascular injury. Emerging data suggest that SAA contributes to atherogenesis. SAA mRNA and protein have been detected in atherosclerotic lesions in both humans and mice [109, 117]. In murine models, lentiviral overexpression of Saa1 in ApoE^−^/^−^ mice resulted in persistent Saa elevation, increased leukocyte infiltration, and accelerated plaque development [36]. Even transient Saa elevations were sufficient to enhance atherosclerosis in similar models [150]. Conversely, genetic deletion of Saa1 and Saa2 in LDLR^−^/^−^ mice significantly reduced aortic lesion area [78]. In ApoE^−^/^−^ mice lacking Saa1and Saa2, additional suppression of Saa3 using antisense oligonucleotides further attenuated plaque formation, compared to Saa-sufficient controls [151]. Although the precise mechanisms remain incompletely defined, proposed pathways include vascular inflammation, endothelial dysfunction, impaired HDL function, and release of lipid-free or lipid-poor SAA isoforms [141]. SAA has also been shown to promote foam cell formation via LOX-1-mediated activation of the JNK/NF-κB pathway in macrophages [88].

## SAA and HFpEF

### Clinical evidence linking SAA levels to HFpEF

SAA is a prototypical acute reactant that is markedly upregulated during acute inflammation. Although its elevation is more modest in chronic metabolic disorders, SAA may still exert important biological effects in such contexts. HFpEF, a syndrome characterized by systemic low-grade inflammation, is particularly relevant in this regard. Early studies in newly diagnosed patients with primary hypertension reported that elevated circulating SAA levels were associated with concentric left ventricular remodeling [153]. More recent investigations have identified positive correlations between SAA1 levels and echocardiographic measures of cardiac structure, including interventricular septal thickness and posterior wall thickness particularly in patients with resistant hypertension [172]. Among women with HFpEF and clinical signs of ischemia, 21% exhibited serum SAA concentrations exceeding 1.0 mg/dL, a threshold considered abnormally high [2]. Elevated plasma SAA1 levels have also been reported in HFpEF patients regardless of the presence of coronary microvascular dysfunction or atrial fibrillation [35]. Notably, elevated SAA1 concentrations observed in patients with chronic HFpEF, compared to those with Hypertrophic Cardiomyopathy (HCM), may help identify individuals with a more advanced or progressive disease phenotype [20]. In a hierarchical clustering analysis of HFpEF patients, three distinct phenotypic subgroups were identified, with SAA1 levels significantly elevated in both the inflammatory cluster and the obesity/high-CRP cluster [132]. Taken together, the available data—summarized in Table 2—indicate that SAA is frequently elevated in a subset of HFpEF patients and, as part of the systemic inflammatory milieu, may contribute to disease progression.

**Table 2:** Summary of clinical studies linking SAA and HFpEF

Design & sample size	Patient characteristics	SAA findings	Clinical outcomes	References
Phenogrouping study; Pan-inflammatory (n = 129) Noninflammatory (n = 83) and obese high CRP (n = 89)	All patients: median age 69 years; 47% male; predominantly White (91%). Patients had advanced HFpEF with high prevalence of hypertension (85%), hyperlipidemia (73%), obesity (median BMI 33.5), diabetes mellitus (38%), COPD (16%), atrial fibrillation (48%), and previous smoking (58%). NYHA functional class III/IV was present in 54% of patients. Median left ventricular ejection fraction (LVEF) was 60% (IQR 56–66)	Elevated SAA along with CRP and IL-6 in obese HFpEF phenogroup	Unique obesity-inflammation phenotypes exist in HFpEF and are associated with differences in comorbidity burden, HFpEF severity, and fibrosis	[130]
Proteomic study; Acute HFpEF (n = 8) Chronic HFpEF (n = 9) HCM (n = 14)	Chronic HFpEF group: mean age 64.6 ± 10.6 years; 66.6% male; mean BMI 28.0 ± 2.5; diabetes prevalence 22.2%; NYHA class I/II; mean LVEF 57.4 ± 8.5%	SAA1 was perturbed in chronic HFpEF compared to HCM with a substantial fold change	New potential protein markers were identified for different HFpEF forms, including LRG1, SAA1 and ITIH3	[19]
Proteomic study; CMD (n = 2) HFpEF (n = 3) CH (n = 3) ACH (n = 5)	All patients: median age 57 years; 83% female; 61% African American; mean LVEF 64 ± 7%; mean BMI 39 ± 11	Serum SAA levels are elevated in patients with HFpEF, irrespective of the presence of AF or CMD	Identified mechanistic pathways and novel circulating biomarkers—including SAA1, LRG1, APOC3, LCP1, PON1, and C1S—linking AF, CMD, and HFpEF, highlighting their translational potential	[34]
Prospective cohort; Women with signs and symptoms of ischemia, no obstructive coronary artery disease and preserved left ventricular ejection fraction (N = 390)	All patients were women; mean age 56 ± 11 years; 4% had diabetes; 13% had dyslipidemia; 17%had a family history of premature CAD; 6% were current smokers; mean BMI 30 ± 7; 3% had chronic kidney disease; 11% had a history of malignancy; and 9% had a history of autoimmune disease	21% of patients had SAA > 1.0 mg/dL, values that are considered abnormally high	In women with signs and symptoms of ischemia, non-obstructive CAD and preserved EF, elevated IL-6 predicted HF hospitalization and all-cause mortality, while SAA level was only associated with all-cause mortality	[2]

*CRP* C-reactive protein, *COPD* chronic obstructive pulmonary disease, *HCM* hypertrophic cardiomyopathy, *CMD* coronary microvascular disease, *CH* CMD and HFpEF, *ACH* atrial fibrillation, CMD and HFpEF

Despite these associations, current human studies are constrained by small sample sizes, predominantly cross-sectional designs, and substantial heterogeneity in comorbidities and demographics. Moreover, variability in SAA quantification methods affects comparability across studies. These limitations highlight the need for prospective cohort and interventional studies that incorporate serial SAA measurements to better define its role in HFpEF onset and progression. Although direct causal evidence is limited, its established pro-inflammatory properties suggest potential roles involving inflammation, endothelial dysfunction, and myocardial fibrosis in HFpEF.

### Potential pathogenetic role of SAA in cardiometabolic HFpEF

#### Inflammation-induced endothelial dysfunction

HFpEF is increasingly recognized as a systemic disorder in which metabolic-related immune and vascular dysfunction plays a central pathogenic role [4, 9, 97, 102]. In individuals with metabolic comorbidities such as obesity, type 2 diabetes, and metabolic syndrome, chronic low-grade inflammation and endothelial impairment synergistically promote coronary microvascular rarefaction and impair NO-dependent vasodilation [102]. These vascular changes compromise myocardial perfusion and oxygen delivery, fostering a state of mechano-energetic uncoupling. Concurrently, cardiomyocyte metabolic flexibility is diminished, with a shift from fatty acid oxidation toward less efficient glucose utilization, mitochondrial dysfunction, and accumulation of toxic lipid intermediates [112]. Within this pathophysiological landscape, SAA may serve as a mediator linking metabolic inflammation to microvascular and myocardial dysfunction. As an acute-phase protein markedly upregulated in metabolic disorders, SAA can act on endothelial cells, vascular smooth muscle cells, and infiltrating immune cells to enhance pro-inflammatory signaling, oxidative stress, and extracellular matrix remodeling.

**Fig. 2:**
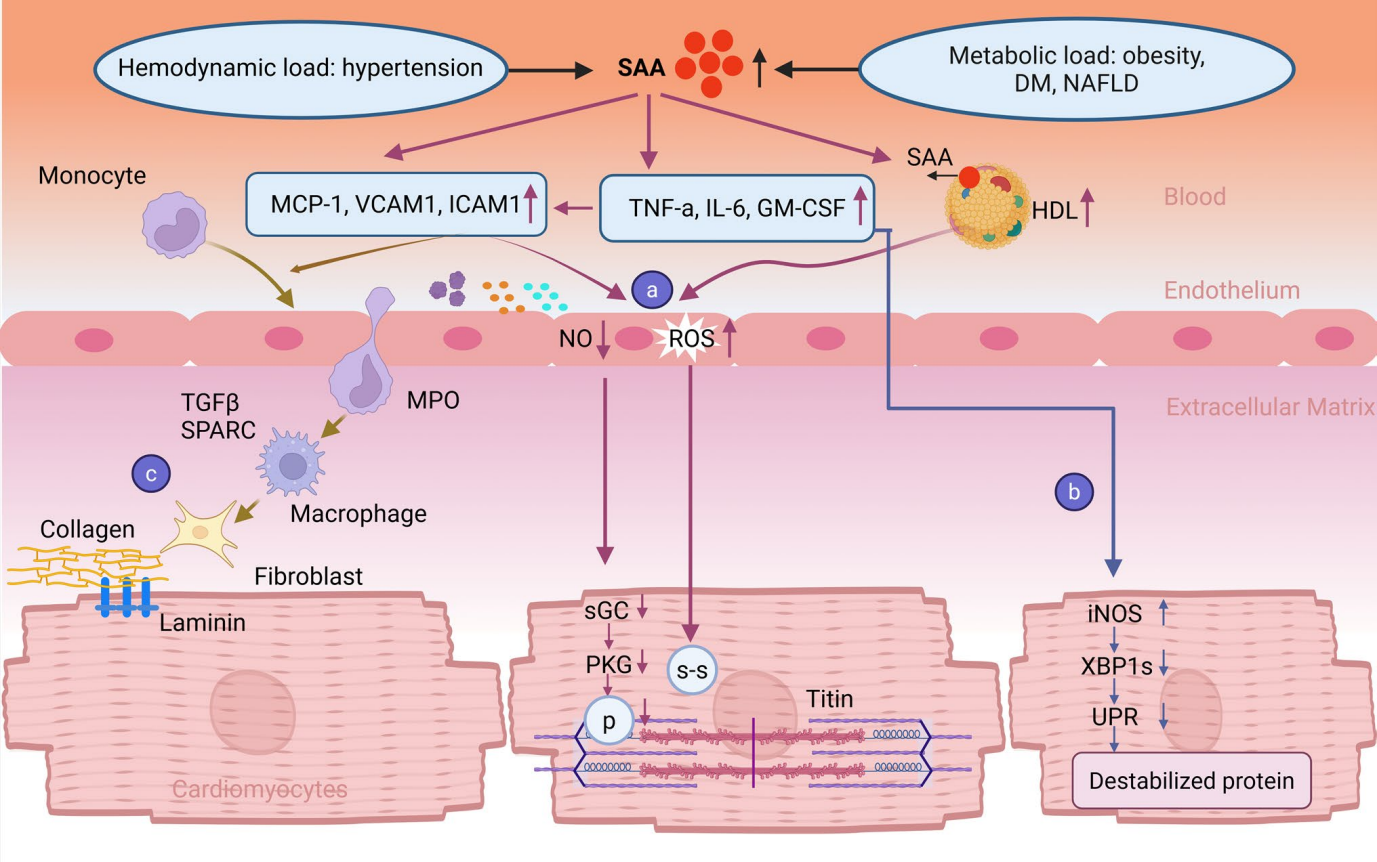
Speculated molecular pathways involving SAA in HFpEF. This figure illustrates the hypothesized mechanisms by which SAA contributes to the development of HFpEF. Both hemodynamic stress (e.g., hypertension) and metabolic load (e.g., obesity, diabetes, NAFLD) can upregulate circulating SAA levels. A central mechanism involves the induction of vascular inflammation through the upregulation of endothelial adhesion molecules (e.g., VCAM1, ICAM1) and proinflammatory cytokines (e.g., TNF-α, IL-6, GM-CSF). **a** Monocyte recruitment via VCAM1 and ICAM1, along with SAA-enriched HDL, contributes to reduced nitric oxide (NO) bioavailability and increased reactive oxygen species (ROS) production in endothelial cells. Myeloperoxidase (MPO) released by activated macrophages further amplifies oxidative stress and endothelial dysfunction. Diminished NO levels attenuate the activity of soluble guanylate cyclase (sGC) and protein kinase G (PKG), leading to titin hypophosphorylation, while elevated ROS induces disulfide bond formation within titin. Both modifications increase cardiomyocyte stiffness (purple). **b** SAA-induced inflammatory cytokines promote systemic inflammation and may upregulate inducible nitric oxide synthase (iNOS) expression in cardiomyocytes. This suppresses the activation of IRE1α, reduces levels of spliced XBP1, and disrupts the adaptive unfolded protein response (UPR), thereby promoting proteostatic stress (blue). **c** Furthermore, monocytes infiltrate the myocardium and differentiate into macrophages, which secrete profibrotic mediators such as transforming growth factor-β (TGF-β) and secreted protein acidic and rich in cysteine (SPARC). These mediators activate fibroblasts and promote collagen synthesis, contributing to extracellular matrix remodeling (gold). This figure integrates current evidence and hypothetical mechanisms to highlight the multifaceted role of SAA in HFpEF pathogenesis

Among these processes, endothelial dysfunction represents a pivotal determinant of vascular pathology. In HFpEF, metabolic stress-induced SAA1 may contribute to endothelial dysfunction by directly or indirectly enhancing the expression of vascular adhesion molecules. Experimental evidence demonstrates that SAA stimulation markedly upregulates the expression of adhesion molecules in vascular endothelial cells, including vascular cell adhesion molecule 1 (VCAM-1), intercellular adhesion molecule 1 (ICAM-1), and E-selectin (SELE) [21, 82, 83]. In addition, as illustrated in Fig. 1, SAA induces the production of pro-inflammatory cytokines such as IL-6 and TNF-α, which are consistently elevated in patients with HFpEF and further augment endothelial adhesion molecule expression. Increased adhesion molecules not only promote monocyte recruitment but also reduce NO bioavailability and enhance reactive oxygen species (ROS) production [120] (Fig. 2, panel a). Together, these alterations accelerate endothelial dysfunction and drive disease progression. Moreover, SAA and its downstream inflammatory mediators may exacerbate systemic and cardiac inflammation in HFpEF and induce iNOS expression in cardiomyocytes, ultimately impairing the unfolded protein response (UPR) and promoting cytosolic accumulation of misfolded proteins [120] (Fig. 2, panel b).

Beyond promoting the expression of endothelial adhesion molecules, SAA1 may exacerbate endothelial dysfunction in HFpEF through its adverse effects on HDL functionality. During inflammation, SAA can constitute up to 80% of HDL’s apolipoprotein content. In HFpEF patients, elevated circulating SAA may alter HDL composition, diminish its anti-inflammatory and antioxidant capacity [23, 54, 138, 152]. Mechanistically, SAA-enriched HDL promotes vascular inflammation via TLR2 and TLR4 activation in vascular smooth muscle cells, which induces MCP-1 production [138]. Moreover, it may contribute to endothelial injury by reducing NO bioavailability and increasing reactive oxygen species (ROS) production [174] (Fig. 2, panel a). Together with the upregulation of adhesion molecules, these effects may converge to accelerate vascular dysfunction in HFpEF.

Persistent low-grade inflammation is considered a major driver of immune dysregulation in HFpEF, facilitating aberrant recruitment and infiltration of immune cells. Metabolic stress-induced SAA1 may further exert chemotactic effects via receptors such as FPRL1 and CLA-1, thereby inducing endothelial cells to secrete a broad spectrum of chemokines, including C–C motif chemokine ligand 2(CCL2/MCP-1), CCL5, CXCL1–3, CXCL8 and CXCL10 [82, 89, 90, 114]. MCP-1 promotes the infiltration of monocytes and macrophages into inflamed tissues, amplifying local immune responses and fostering myocardial fibrosis [34, 45] (Fig. 2, panel c). Notably, patients with HFpEF exhibit elevated circulating MCP-1 levels and increased numbers of pro-inflammatory monocytes, with myocardial biopsies revealing marked infiltration of activated macrophages and monocytes [45, 52, 63]. These observations suggest that SAA1, through the induction of chemokines such as MCP-1, may contribute to immune cell activation and maladaptive cardiac remodelling in HFpEF.

#### Induction of myocardial fibrosis

The pathophysiology of HFpEF involves complex, multi-organ interactions, among which myocardial fibrosis plays a central role by contributing to diastolic dysfunction and increased myocardial stiffness [85]. In patients with HFpEF, excessive ECM remodeling, which is characterized by collagen accumulation, cross-linking, and reduced myocardial compliance, correlates strongly with echocardiographic and hemodynamic indices of diastolic dysfunction [41, 74, 100]. As depicted in Fig. 1, inflammatory mediators such as IL-1, IL-6, and TNF-α-activated downstream of SAA-receptor signaling—are closely linked to profibrotic signaling cascades in HFpEF [103, 115]. Beyond cytokine-driven inflammation, SAA has been shown to directly influence fibroblast activation and proliferation. SAA stimulates the proliferation of murine cardiac fibroblasts in vitro [56]. In vivo, genetic deletion of Saa1 has been reported to decrease the expression of collagen I, collagen IV, and fibronectin in pressure overload preclinical models, while Saa1 silencing mitigated the transformation of cardiac fibroblasts into myofibroblasts following TGF-β stimulation [164]. These findings suggest that SAA may modulate cardiac fibrogenesis in HFpEF through both inflammatory and direct cellular mechanisms, although its precise mechanistic role in myocardial fibrosis remains to be elucidated.

### SAA in aging- and CKD-associated HFpEF

Multiple pathophysiological factors contribute to HFpEF, with aging playing an important role [13, 50]. Exploring molecular insights into age-related changes in myocardial function, a key factor influencing global cardiovascular reserve. Aging is associated with a systemic pro-inflammatory state, termed “inflammaging,” which can impair the function of multiple organs even in the absence of specific disease [44]. Indeed, several cross-sectional studies have demonstrated that advancing age correlates with elevated circulating levels of inflammatory markers, including TNF-α, IL-6, IL-18, and monocyte chemoattractant protein-1 [25, 113, 119]. In addition, one research indicated that SAA levels have been shown to rise with age even in the absence of overt infection, potentially reflecting a chronic inflammatory state [84]. In another study, levels of the inflammatory marker SAA increased significantly with age in humans or mice without metabolic syndrome [40]. Elevated SAA in aging as well as in metabolic syndrome, may trigger coronary microvascular endothelial dysfunction through the induction of inflammatory cytokines and adhesion molecules. This process ultimately promotes interstitial fibrosis and increases cardiomyocyte stiffness, leading to enhanced left ventricular diastolic stiffness and the onset of heart failure. Cardiometabolic HFpEF is frequently accompanied by metabolic comorbidities such as obesity, diabetes, MASLD—conditions that are well-recognized drivers of accelerated cardiovascular aging. Consequently, cardiometabolic HFpEF can be regarded as a paradigm of inflammation-driven cardiac aging. Within this framework, SAA may serve as a common mechanistic contributor across these two HFpEF phenotypes.

CKD-associated HFpEF is strongly linked to systemic inflammation, uremic toxin accumulation, oxidative stress, and profound endothelial dysfunction. In this context, CKD patients often exhibit markedly elevated circulating SAA levels [26, 65, 71, 142], and a recent meta-analysis demonstrated a positive, linear association between SAA levels and the risks of all-cause and cardiovascular mortality in this population [93]. Diabetic nephropathy, the leading cause of CKD worldwide, highlights this association. An immunohistochemical study demonstrated widespread SAA protein deposition in the glomeruli and tubulointerstitium of both diabetic nephropathy patients and mouse models [3]. Podocytes were further identified as potential responder cells in SAA-driven renal inflammation. Experimental data also support a pathogenic role of SAA, as ApoE−/− mice exposed to SAA developed renal injury within 4 weeks, characterized by increased plasma urea, urinary protein, oxidized lipids, urinary kidney injury molecule (KIM)-1, and elevated cytokines and chemokines in kidney tissue compared with controls [18]. Beyond renal injury, SAA also impairs vascular homeostasis by inducing aortic endothelial dysfunction, as evidenced by upregulation of VCAM-1 and MCP-1 expression and suppression of cyclic guanosine monophosphate (cGMP) signaling [18]. By concurrently promoting renal inflammation and vascular dysfunction, SAA emerges as a potential pathogenic mediator linking CKD and CKD-associated HFpEF.

## Clinical applications targeting SAA in cardiometabolic diseases

SAA levels rise rapidly in response to infection or trauma and are considered a highly sensitive marker of acute inflammation [16, 70, 107, 116]. While SAA is widely used as a general inflammatory marker in murine studies, CRP remains the more commonly employed biomarker in human clinical research. This is partly attributable to technical challenges associated with SAA, including issues related to purification, antibody development, assay standardization, and limited analytical sensitivity. It also underscores the need for caution when extrapolating preclinical findings involving SAA to human HFpEF. Notably, emerging evidence suggests that circulating SAA may outperform high-sensitivity CRP (hsCRP) in predicting cardiovascular events [30, 70, 77]. In a prospective cohort of 705 women undergoing coronary angiography, elevated plasma SAA was independently associated with adverse cardiovascular outcomes—including nonfatal myocardial infarction, stroke, heart failure, and thrombosis—over 3 years [70]. Similar findings have been reported in postmenopausal women and in patients with suspected or confirmed coronary artery disease (CAD) [129, 149]. Although SAA1 levels are elevated in HFpEF patients, whether SAA1 levels offer incremental prognostic value beyond established markers in HFpEF remains an open question.

**Fig. 3:**
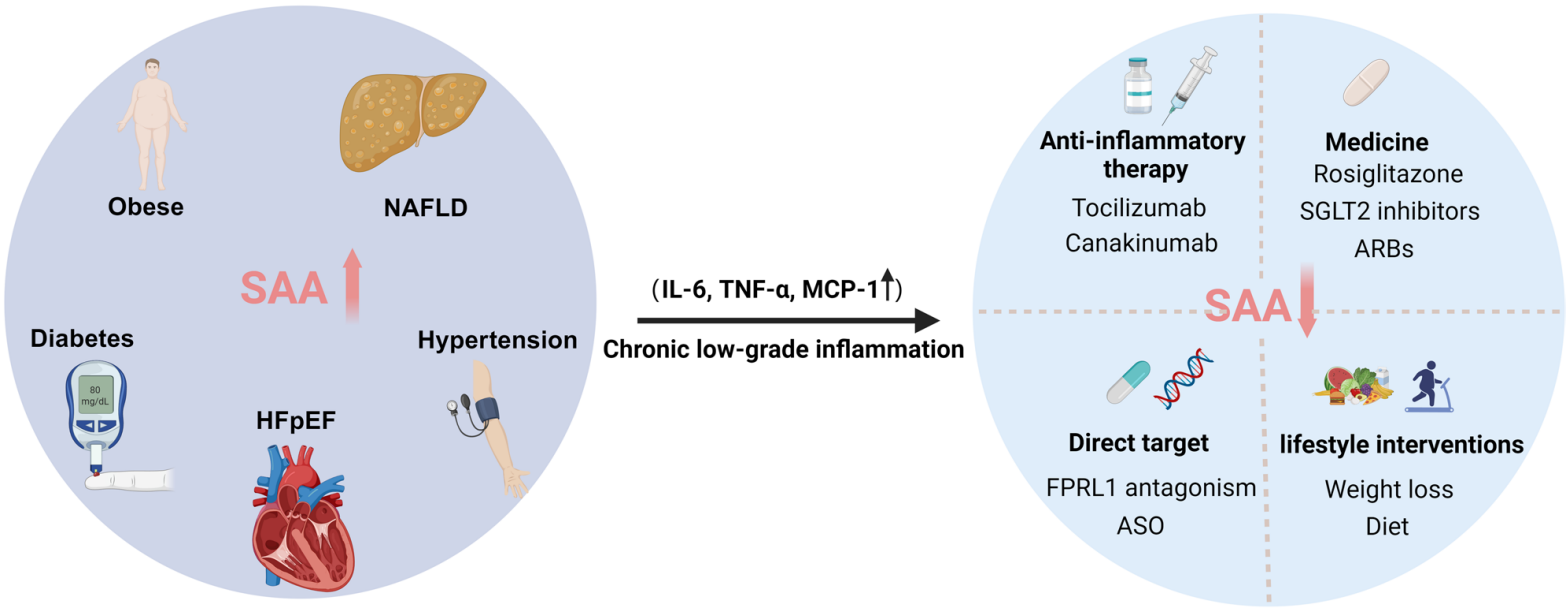
Illustrative depiction summarizing the role of SAA in cardiometabolic diseases and potential therapeutic strategies. SAA is elevated in metabolic conditions such as obesity, type 2 diabetes, NAFLD, hypertension, and HFpEF, all of which are characterized by chronic low-grade inflammation. In this context, SAA may serve both as a biomarker and as a mediator of disease progression. Therapeutic strategies targeting SAA include: (1) Direct inhibition of SAA production and signaling, such as through antisense oligonucleotides for suppressing its synthesis and FPRL1 antagonists for blocking SAA-mediated signaling pathways; (2) Inhibition of upstream cytokines involved in SAA induction, including IL-6 (e.g., tocilizumab) and IL-1β (e.g., canakinumab); (3) Pharmacological agents with indirect anti-inflammatory effects, including antidiabetic drugs (e.g., rosiglitazone, SGLT2 inhibitors) and angiotensin receptor blockers (ARBs); (4) Lifestyle interventions (e.g., weight loss, dietary modifications) that may reduce systemic inflammation and SAA levels

Therapeutically, targeting SAA represents a promising strategy to modulate inflammation in cardiometabolic disease (Fig. 3). As a hepatocyte-derived acute-phase protein primarily regulated by IL-6, IL-1β, and TNF-α, SAA expression can be indirectly suppressed by anti-inflammatory therapies. Tocilizumab, an IL-6 receptor antagonist, has been shown to reduce circulating SAA levels and has been employed in amyloidosis and rheumatoid arthritis [53, 61, 118]. More broadly, the CANTOS trial established that IL-1β blockade canakinumab can reduce cardiovascular events by dampening systemic inflammation [128], supporting the therapeutic value of anti-cytokine strategies. In parallel, direct inhibition of SAA signaling—such as via FPRL1 antagonism—has shown anti-inflammatory and vasoprotective effects in preclinical studies [17]. RNA-based approaches, including ASO, have shown promise in targeting SAA isoforms and may offer tissue-specific modulation [81, 151]. Beyond pharmacological approaches, Lifestyle interventions—including weight loss, exercise, and dietary modification—also lower SAA indirectly by mitigating upstream metabolic triggers such as insulin resistance and adiposity.

Clinical studies suggest that some antidiabetic agents lower serum SAA levels in patients with T2DM, yet the extent to which this effect is independent of glycemic regulation remains unclear. One study reported rosiglitazone significantly reduced serum SAA levels as early as 2 weeks into treatment, and this reduction was sustained over 12 weeks. In this study, the change in SAA levels after 12 weeks was significantly correlated with changes in fasting glucose levels [104]. This association may be explained by the fact that reductions in fasting blood glucose are relatively modest during the initial 2–4 weeks of rosiglitazone treatment, whereas stable glycemic control is typically achieved only after 8–12 weeks. Therefore, the early decline in SAA cannot be simply interpreted as a consequence of improved glycemic control. However, another study demonstrated that low-dose rosiglitazone (2 mg) exerted a marked anti-inflammatory effect over 6 weeks in T2DM patients, reducing SAA levels by 29% while increasing adiponectin and decreasing resistin levels, without any detectable changes in plasma glucose, free fatty acids (FFA), or insulin concentrations [49]. This effect may be attributable to the fact that SAA1 and SAA2 are potential downstream targets of peroxisome proliferator-activated receptor gamma (PPARγ) [167]. Similarly, in non-diabetic patients with symptomatic carotid stenosis, rosiglitazone treatment (4 mg) for 4 weeks significantly reduced serum SAA levels by 33%, without altering glucose or insulin levels [111]. These findings strengthen the rationale for considering SAA as a potential direct therapeutic target in diabetes, rather than merely a downstream marker of improved glucose metabolism. Recent years have witnessed growing interest in the pleiotropic effects of GLP-1RAs and SGLT2 inhibitors. Beyond improving glycemic control, these drugs have been shown to alleviate diabetic complications and cardiovascular disease by reducing inflammation and oxidative stress [12, 106]. To date, direct clinical evidence demonstrating modulation of SAA levels or activity by these drug classes remains limited. However, in ApoE−/− mice fed a Western diet for 20 weeks, oral treatment with empagliflozin for 8 weeks significantly reduced the area of atherosclerotic plaques in the aortic arch and valve, and also significantly decreased circulating Saa levels from 24.5 ± 3.6 μg/ml to 16.2 ± 3.9 μg/ml [55]. While no direct preclinical evidence currently demonstrates that GLP-1RAs reduce circulating SAA, both animal studies and clinical data indicate their ability to suppress proinflammatory cytokines such as IL-6, TNF-α, and IL-1β [11]. These cytokines are potent inducers of SAA in hepatic and adipose tissues, suggesting that GLP-1RAs may theoretically modulate SAA expression. Future preclinical and clinical investigations related to GLP-1RAs and SGLT2 inhibitors are warranted to monitor less-studied inflammatory mediators such as SAA, which could broaden therapeutic strategies targeting SAA in cardiometabolic diseases.

## Conclusion and future perspectives

SAA has emerged as a promising inflammatory biomarker and potential driver in cardiometabolic HFpEF. However, key mechanistic gaps remain. We highlight four high-priority areas for future investigation. First, clarifying whether SAA is a causal driver or merely a bystander in HFpEF will require prospective cohorts with serial SAA measurements and Mendelian randomization analyses, complemented by cardiac tissue profiling using spatial transcriptomics or proteomics. Second, the downstream signaling pathways of SAA remain poorly understood. Single-cell or spatial omics, combined with cell-specific gene editing in mouse models, could help define receptor–cell interactions in cardiomyocytes, fibroblasts, and endothelial cells. Third, given HFpEF heterogeneity, future trials should stratify patients based on comorbidity clusters and inflammatory profiles through multicenter cohorts, integrating multi-omics data to refine patient classification. Fourth, standardization across all stages of SAA measurement—from sampling to interpretation—is essential for clinical application. This requires reference materials, harmonized platforms, validated ranges, and strong quality control, following models established for CRP and NT-proBNP. To accelerate progress, early-phase interventions—such as SAA-neutralizing antisense oligonucleotides in well-characterized HFpEF subgroups—should be prioritized. These priorities, together with other unresolved issues, are summarized in Table 3. Moving forward, a coordinated research agenda that bridges mechanistic discovery and early clinical translation is urgently needed to realize the full diagnostic and therapeutic potential of SAA in HFpEF.

**Table 3:** Key mechanistic and translational gaps in understanding SAA in HFpEF

Unresolved questions
1. What is the spatial distribution of SAA in cardiac tissue, including its localization to cardiomyocytes, endothelial cells, and fibroblasts?
2. Which specific receptors mediate SAA signaling across different cardiac cell types, and how do their downstream pathways differ?
3. Does SAA exert cell-type–specific effects on endothelial cells, fibroblasts, and cardiomyocytes in the context of HFpEF?
4. Through which molecular mechanisms does SAA contribute to extracellular matrix remodeling and fibroblast activation in the myocardium?
5. How does SAA-enriched HDL alter endothelial function and vasoprotective properties in cardiometabolic HFpEF?
6. Is SAA an initiating factor in HFpEF pathogenesis or a secondary amplifier of existing inflammatory pathways?
7. Can the inclusion of SAA in a multimarker panel improve risk stratification and prognostic accuracy in cardiometabolic HFpEF?
8. Do rising SAA levels precede clinical progression of HFpEF, and can they serve as an early biomarker of disease development?
9. Is SAA similarly elevated in non-cardiometabolic HFpEF subtypes such as those associated with aging, coronary artery disease (CAD), or pulmonary hypertension (pHTN)?
10. Can therapeutic targeting of SAA (e.g., antisense oligonucleotides, receptor antagonists) reduce myocardial inflammation and improve cardiac function in HFpEF?
11. Do SGLT2 inhibitors modulate SAA expression in HFpEF, and does this contribute to their observed anti-inflammatory and cardioprotective benefits?

## Data Availability

No data was used for the research described in the article.

## Abbreviations

ARB: Angiotensin receptor blocker
ASO: Antisense oligonucleotide
CAD: Coronary artery disease
CCL2: C–C motif chemokine ligand 2
CRP: C-reactive protein
ERK: Extracellular signal-regulated kinase
ECM: Myocardial extracellular matrix
FPRL1: Formyl peptide receptor-like 1
G-CSF: Granulocyte colony-stimulating factor
GM-CSF: Granulocyte–macrophage colony-stimulating factor
GQAS: Global quality assessment schemes
HDL: High-density lipoproteins
HFpEF: Heart failure with preserved ejection fraction
HFrEF: Heart failure with reduced ejection fraction
ICAM-1: Intercellular adhesion molecule 1
IL-6: Interleukin-6
MAPK: Mitogen-activated protein kinase
MASLD: Metabolic dysfunction associated steatotic liver disease
MCP-1: Monocyte chemotactic protein-1
M-CSF: Macrophage colony-stimulating factor
MMP: Matrix metalloproteinase
MPO: Myeloperoxidase
NAFLD: Non-alcoholic fatty liver disease
NO: Nitric oxide
pHTN: Pulmonary hypertension
SAA: Serum amyloid A
SELS: Selenoprotein S
TLR2: Toll-like receptor 2
TNF-α: Tumor necrosis factor-α
T2DM: Type 2 diabetes mellitus
VCAM-1: Vascular cell adhesion molecule 1
WAT: White adipose tissue
